# Introduction to the special edition of the Proceedings of the Fifth Annual Symposium of the Michael G. DeGroote Institute for Pain Research and Care, held November 2018

**DOI:** 10.1080/24740527.2019.1630266

**Published:** 2019-07-30

**Authors:** D. Norman Buckley

**Affiliations:** Michael G. DeGroote School of Medicine, Department of Anesthesia, McMaster University, Hamilton, Ontario, Canada

It is with great pride and pleasure that I am writing to introduce this special edition of the *Canadian Journal of Pain*. We intend with the proceedings of this edition to both highlight the potential value of persistent postsurgical pain as a conceptual model for studying chronic pain and introduce to many of you the work supported by the endowment from Michael G. DeGroote that created the Institute for Pain Research and Care at McMaster University.

Many of you will be aware of the substantial gift from Mr. DeGroote in 2003 that led to the naming of McMaster Medical School as the Michael G. DeGroote School of Medicine at McMaster University. Included in this gift were several specifically directed endowments, one of which created the Michael G. DeGroote Institute for Pain Research and Care. This was created in part to support ongoing search for a cure for the devastating poststroke central pain that Mr, DeGroote experiences but also to further work in the area of understanding and treating pain in general. This work includes basic science, clinical science in medicine, surgery and the behavioral sciences, knowledge synthesis and translation, and advocacy for pain-related health care policy.

Since 2010, knowledge synthesis and translation has been supported through the work of the Michael G. DeGroote National Pain Centre, which has as its mission the creation and dissemination of best practice knowledge for pain care and currently holds the copyright and responsibility for updating and disseminating the *Canadian Opioid Guideline*. This was originally created in 2010 by the National Opioid Use Guideline Group, an ad hoc committee of the Federation of Medical Regulatory Authorities of Canada, and was revised and updated in 2017.

In support of the *Canadian Opioid Guideline*, the National Opioid Use Guideline Group created the national faculty that continues to this day coordinated by the National Pain Centre and works to enhance the dissemination of the *Opioid Guideline* to patients and the public, prescribers, pharmacists, and policymakers.

Advocacy is carried out through the activities of the Canadian Pain Care Forum, supported by the Institute for Pain Research and Care and composed of over 70 volunteers from across the country representing more than 45 organizations, including patient advocacy groups, regulators, educators, and health care professionals along with members of the health care bureaucracy and the community at large. This group meets quarterly to provide a forum for coordinating efforts to inform and pursue the necessary health care policies and actions to ensure best quality, safe pain care in Canada.

In 2014 the Institute established a program of research that uses the problem of persistent postsurgical pain as a means of conceptually approaching the study of chronic pain. The idea was to use the McMaster history of interdisciplinary collaboration to ensure that the topic of pain was explored from as many vantage points as possible—anesthesia, surgery, peri-operative medicine, nursing, rehabilitation science, psychology, and behavioral sciences—and to make use of the unique resources at McMaster, including the experience with application of the tools of clinical epidemiology, large-scale clinical trials, and excellent clinical care. The Institute membership is composed of researchers based within McMaster’s Faculty of Health Sciences, and the budget supports a mix of competitive awards, research staff to enable clinician led research, and the scientific and administrative leadership. Our Scientific Advisory Board consists of national and international leaders in the field of pain research and care: Manon Choiniere, Ian Gilron, Joel Katz, Henrik Kehlet, and Mark Lema. This group adjudicates our annual awards as well as advises on the research direction and activities of the Institute. I particularly thank Professor Henrik Kehlet for his kindness in offering an overview of the material in this special issue.

Each year we hold a symposium that features invited keynote speakers, members of our Scientific Advisory Board, and the work of recipients of funding through our graduate scholarships, postdoctoral fellowships, and seed grant competitions. Keynote speakers have included Jane Quinlan (in the most recent symposium and reported in this issue), Martin Angst, Mathew Chan, Luda Diatchenko, Mike Salter, and Hance Clarke. Their work covers a broad spectrum of pain research and has informed our own discussions locally as well as those on the larger stage. The annual meeting, which is held in early November, is open to the local, regional, and national pain community interested better understanding and managing persistent chronic postsurgical pain.

Our intent with this special issue, which we hope will be emulated by other Canadian pain research institutes, is to highlight our work by publishing the proceedings of the Fifth Annual Symposium of the Institute. We believe that the *Canadian Journal of Pain*, the official journal of our national professional association, the Canadian Pain Society, is the ideal forum in which to accomplish our goal of furthering pain research across Canada. I do hope that you will appreciate the material that appears in this issue and look forward to hearing from you in the future.

